# Supplementary material for: Unveiling Mechanisms of SEI Formation and Sodium Loss in Sodium Batteries via Interface Reactor Sampling
Source: Adv Sci (Weinh). 2026 Jul 31;13(59):e76808. doi: 10.1002/advs.76808 (PMC13426102; doi:10.1002/advs.76808)
Supplement: Supplementary file 1 [file ADVS-13-e76808-s001.docx]

**Supplementary Information**

**for**

**“Unveiling Mechanisms of SEI Formation and Sodium Loss in Sodium Batteries via Interface Reactor Sampling”**

Zhoulin Liu^abcd^, Ziliang Wang^b*^, Zherui Chen^e^, Jianchun Sha^f^, Fengzijun Pan^g^, Pingyang Zhang^b^, Yinghe Zhang^acd*^

^a^ School of Science, Harbin Institute of Technology, Shenzhen 518055, Guangdong, P.R. China

^b^ National Engineering Laboratory for Reducing Emissions from Coal Combustion, Engineering Re-search Center of Environmental Thermal Technology of Ministry of Education, Shandong Key Laboratory of Green Thermal Power and Carbon Reduction, Shandong University, Jinan, Shandong 250061, P. R. China

^c^ The Key Laboratory of Advanced Materials AI Manufacturing and Micro-Nano Processing, Harbin Institute of Technology, Shenzhen 518055, China

^d^ Shenzhen Key Laboratory of Advanced Functional Carbon Materials Research and Comprehensive Application, Harbin Institute of Technology, Shenzhen 518055, China

^e^ College of Applied Sciences, Shenzhen University, Shenzhen 518060, P.R. China

^f^ Key Lab of Electromagnetic Processing of Materials, Ministry of Education, Northeastern University, Shenyang 110819, PR China

^g^ School of Transportation Science and Engineering, Harbin Institute of Technology, Harbin 150090, P.R. China

*Corresponding authors. E-mail addresses: zwang2022@sdu.edu.cn (Ziliang Wang), zhangyinghe@hit.edu.cn (Yinghe Zhang).

**Contents**

[Supplementary Note S1: Charge-aware Neuroevolution Potential	3](#_Toc221021046)

[Supplementary Note S2: SNES-based Multi-loss Training Algorithm and the nep.in Input File	4](#_Toc221021047)

[Supplementary Note S3: Vasp Input Parameters	5](#_Toc221021048)

[Supplementary Note S4: Validation of Structural Convergence via Net Force Analysis	6](#_Toc221021049)

[Supplementary Note S5: Definition of Radial and Angular Descriptors in NEP	7](#_Toc221021050)

[Supplementary Note S6: Initial Training Set Construction and Physics-Guided Active Learning	8](#_Toc221021051)

[Supplementary Note S7: Training Set Composition	9](#_Toc221021052)

[Supplementary Note S8: Stability Validation of Large-Scale Simulations	10](#_Toc221021053)

[Supplementary Note S9: Comparison of Force Prediction Accuracy between ReaxFF and DFT Results.	11](#_Toc221021054)

[Supplementary Note S10: DFT- and NEP-calculated Energy Barriers for EC Ring-opening	12](#_Toc221021055)

[Supplementary Note S11: Calculation results for the melting point of metallic sodium.	13](#_Toc221021056)

[Supplementary Note S12: Evolution of Reactive Species in Sodium Electrodes with Various Electrolytes	14](#_Toc221021057)

[Supplementary Note S13: Surface Energies of Low-index NaF.	18](#_Toc221021058)

[Supplementary Note S14: Thermodynamic Analysis of CO vs CO₂ Formation from EC Decomposition	19](#_Toc221021059)

[Supplementary Note S15: Comparison of CO and CO_2_ evolution in the EC system during MLMD simulations	19](#_Toc221021060)

[Supplementary Note S16: Influence of Concentration on the Composition of Solvated Species	20](#_Toc221021061)

[Supplementary Note S17: Temperature and Concentration Dependence of Electrolyte Transport Properties	20](#_Toc221021062)

[Supplementary Note S18: Role of Aggregates (AGGs) in Non-Uniform SEI Growth	21](#_Toc221021063)

[Supplementary Note S19: Well-Tempered Metadynamics Simulations	22](#_Toc221021064)

[Supplementary Note S20: Structural Evolution of the Substrate during Sodium Storage	24](#_Toc221021065)

[Supplementary Note S21: Evolution of Electrolyte Consumption during the Charging Process.	24](#_Toc221021066)

[Supplementary Note S22: Effect of Pore Size and Spatial Confinement on Sodium Storage	25](#_Toc221021067)

[Supplementary Note S23: X-ray Photoelectron Spectroscopy (XPS) Measurements.	26](#_Toc221021068)

[Supplementary Note S24: Gas Chromatography (GC) Measurements.	26](#_Toc221021069)

[REFERENCES	27](#_Toc221021070)

## Supplementary Note S1: Charge-aware Neuroevolution Potential

The development of the Charge-aware Neuroevolution Potential^1^ (qNEP) is based on the GPUMD 4.3 software package^2^. The implementation follows the charge-optimized framework proposed by Song et al.^3^

The core of this framework lies in its use of a single neural network model for each element to simultaneously predict the local atomic energy $E_{i}^{\mathrm{loc}}$ and the atomic charge $q_{i}$, eliminating the need for any reference charge data derived from first-principles calculations. To ensure conservation of the total system charge Q (typically zero), the raw charges output by the network are corrected as follows:

$${\overset{^}{q}}_{i}=q_{i}-\frac{1}{N}(\sum_{j=1}^{N} q_{j}-Q)$$

where ${\overset{^}{q}}_{i}$ is the final corrected atomic charge and *N* is the total number of atoms. These corrected charges are then used to compute the electrostatic energy $E^{\mathrm{elec}}$ of the system via the Ewald summation method. The total energy of the system is consequently given by the sum of the local and electrostatic contributions:

$$E^{\mathrm{tot}}=\sum_{i}^{N} E_{i}^{\mathrm{loc}}+E^{\mathrm{elec}}(\mathbf{R},\mathbf{q})$$

The potential is trained by minimizing a loss function that incorporates errors only in the total energy and atomic forces with respect to density functional theory (DFT) reference values:

$$\mathrm{Loss}=p_{e}{(\frac{E^{\mathrm{DFT}}-E^{\mathrm{tot}}}{N})}^{2}+\frac{p_{f}}{3N}\sum_{i,\beta} {(F_{i,\beta}^{\mathrm{DFT}}-F_{i,\beta}^{\mathrm{tot}})}^{2}$$

Throughout this process, the assignment of atomic charges is not predefined by external data but is implicitly and self-consistently learned through the reproduction of DFT-level energies and forces, thereby enabling effective modeling of long-range electrostatic interactions.

## Supplementary Note S2: SNES-based Multi-loss Training Algorithm and the nep.in Input File

Machine Learning Potential (MLP) is established based on the Neuroevolution Potential (NEP) method, and integrates its unique advanced Separable Natural Evolution Strategy (SNES)^4^. It not only achieves DFT-level accuracy, but also has much higher computational efficiency, enabling the calculation of large systems. This work used the NEP-4 model for potential training^1^. SNES is employed to minimize the loss function and efficiently optimize the free parameters within the NEP model. The total loss function is defined as the weighted sum of several individual losses:

$$\text{L}\text{=}\text{λ}_{\text{1}}\text{L}_{\text{1}}\text{+}\text{λ}_{\text{2}}\text{L}_{\text{2}}\text{+}\text{λ}_{\text{e}}\text{Δ}\text{U}\text{+}\text{λ}_{\text{f}}\text{Δ}\text{F}\text{+}\text{λ}_{\text{v}}\text{Δ}\text{W}\text{ }\text{(5)}$$

Here, $\text{Δ}\text{U}$, $\text{Δ}\text{F}$, and $\text{Δ}\text{W}$ represent the RMSE between the predicted and DFT values for energy, force, and virial. $\text{L}_{\text{1}}$ and $\text{L}_{\text{2}}$ are regularization coefficients, and the $\text{λ}_{\text{e}}$，$\text{λ}_{\text{f}}$，$\text{λ}_{\text{v}}$，$\text{λ}_{\text{1}}$，$\text{λ}_{\text{2}}$ denote the weighting factors assigned to each corresponding term.

The table of hyperparameters is provided,

**Table S1.** Hyperparameters for training of the NEP model

| Hyperparameters | value | Description |
| --- | --- | --- |
| version | 4 | NEP model version (NEP4) |
| zbl | 2 | Outer cutoff for the universal [ZBL](https://gpumd.org/glossary.html#term-ZBL) potential |
| cutoff | 6, 5 | Radial and angular cutoff radii (in Å) |
| n_max | 4, 4 | Number of radial functions for radial and angular descriptors (4 + 1 = 5) |
| basis_size | 8, 8 | Number of basis functions (8 + 1 = 9) |
| l_max | 4, 2, 1 | Maximum angular momentum for 3-body, 4-body, and 5-body terms |
| neuron | 80 | Number of neurons in the hidden layer per element |
| lambda_1 | 0 | L1 regularization weight (disabled) |
| lambda_e | 1 | Energy loss weight |
| lambda_f | 1 | Force loss weight |
| lambda_v | 0.1 | Virial loss weight |
| batch | 5000 | Size of each batch used during the [optimization procedure](https://gpumd.org/potentials/nep.html#nep-optimization-procedure) |
| population | 50 | Size of the population used by the [SNES](https://gpumd.org/glossary.html#term-SNES) algorithm |
| generation | 1,000,000 | Number of generations for the [SNES](https://gpumd.org/glossary.html#term-SNES) algorithm |

All other parameters are set to their default values. However, it should be noted that parameters may vary slightly between different GPUMD software versions.

## Supplementary Note S3: Vasp Input Parameters

Electronic structure and energy calculations were automatically performed using the VASPTool software package.^13^ The table of Parameters for Static Calculations is provided,

Table S1. Parameters for Static Calculations

| **Parameter** | **Value** | **Description** |
| --- | --- | --- |
| ISTART | 0 | Initialization without reading previous wavefunction or charge density files. |
| ICHARG | 2 | Initial charge density is constructed by superposition of atomic charges. |
| PREC | Normal | Standard precision setting influencing the FFT grid density. |
| ENCUT | 600 | Kinetic energy cutoff for the plane-wave basis set, specified in electronvolts. |
| NSW | 0 | Specifies a single-point (static) calculation with no ionic movement. |
| ALGO | F | Electronic minimization using the fast RMM-DIIS algorithm. |
| EDIFF | 1E-06 | Convergence threshold for the electronic energy during self-consistent iterations. |
| LREAL | Auto | Automatic determination for evaluating projection operators in real or reciprocal space. |
| NELM | 120 | Upper limit on the number of electronic self-consistency cycles. |
| NELMIN | 4 | Minimum number of electronic steps required before convergence checks. |
| ISYM | 0 | Turns off all symmetry operations for the calculation. |
| ISMEAR | 0 | Selects Gaussian smearing for partial occupancies. |
| SIGMA | 0.05 | Width of the Gaussian smearing, in units of eV. |
| ISPIN | 1 | Indicates a non-spin-polarized calculation. |
| GGA | PE | Exchange-correlation functional treated with the Perdew-Burke-Ernzerhof generalized gradient approximation. |
| IBRION | -1 | Algorithm flag set for molecular dynamics or static runs (here, static). |
| ISIF | 2 | Calculate the stress tensor and, if relaxation were active, allow cell shape changes. |
| LCHARG | False | Suppresses writing of the CHGCAR file. |
| LWAVE | False | Suppresses writing of the WAVECAR file. |
| IVDW | 12 | DFT-D3 dispersion correction with Becke–Johnson damping |

Additionally, the KPOINTS file was also automatically generated, maintaining a k*a value of 27, which corresponds to a reciprocal space k-point interval of 0.037 Å^−1^.

## Supplementary Note S4: Validation of Structural Convergence via Net Force Analysis

Figure S1. Convergence validation of training set structures via net force analysis.

The net forces of all training configurations are distributed narrowly around zero (mean ≈ 0 meV/Å), confirming that all DFT calculations have fully converged. This validates the structural quality of the training dataset for qNEP model training.

## Supplementary Note S5: Definition of Radial and Angular Descriptors in NEP

In the high-dimensional neural network potential (HDNNP) method proposed by Behler and Parrinello, atom-centered symmetry functions (ACSFs) were first defined and employed as descriptors for atomic environments. The atomic energy $U_{i}(\mathbf{q})$ is expressed as a function of a descriptor vector with *N*_des_ components:

$$U_{i}=\sum_{\mu=1}^{N_{\mathrm{neu}}} w_{\mu}^{(1)}\tanh(\sum_{\nu=1}^{N_{\mathrm{des}}} w_{\mu\nu}^{(0)}q_{\nu}^{i}-b_{\mu}^{(0)})-b^{(1)}$$

Here, tanh(x) serves as the activation function in the hidden layer, $w_{\mu\nu}^{(0)}$ represents the weight matrix connecting the input layer (descriptor vector) to the hidden layer, $w_{\mu}^{(1)}$ denotes the weight vector connecting the hidden layer to the output node (i.e., the energy $U_{i}$, $b_{\mu}^{(0)}$ is the bias vector in the hidden layer, and $b^{(1)}$ is the bias value at the output node $U_{i}$.

Following this approach, the descriptors of NEP model consist of a set of radial and angular components. Among these radial descriptor components $\text{q}$, the sum is defined as all neighboring atoms of atom $\text{i}$ within a certain cutoff distance, resulting in $\text{(}\text{n}_{\text{max}}^{\text{R}}\text{ }\text{+}\text{ }\text{1)}\text{ }$radial descriptor components.

$$\text{q}_{\text{n}}^{\text{i}}\text{=}\sum_{\text{j}\text{≠1}} \text{g}_{\text{n}}\text{(}\text{r}_{\text{ij}}\text{)}\text{ }\text{with}\text{ }\text{0≤}\text{n}\text{≤}\text{n}_{\text{max}}^{\text{R}}\text{ }\text{(1)}$$

The angular components are described using integrated four-body descriptors $\text{q}_{\text{n}\text{l}_{\text{1}}\text{l}_{\text{2}}\text{l}_{\text{3}}}^{\text{i}}$ (where$\text{ }\text{0≤}\text{n}\text{≤}\text{n}_{\text{max}}^{\text{A}}$, and $\text{1≤}\text{l}_{\text{1}}\text{=}\text{l}_{\text{2}}\text{=}\text{l}_{\text{3}}\text{≤}\text{l}_{\text{max}}^{\text{3}\text{b}}$) in Eq. (2), incorporating three-body descriptors $\text{q}_{\text{nl}}^{\text{i}}$ (where $\text{0≤}\text{n}\text{≤}\text{n}_{\text{max}}^{\text{A}}$, and $\text{1≤}\text{l}\text{≤}\text{l}_{\text{max}}^{\text{3}\text{b}}$) in Eq. (3). The difference in positions between two atoms in the equation is calculated by spherical harmonics $\text{Y}_{\text{lm}}\left( \text{θ}_{\text{ij}} \right)$ and $\text{Y}_{\text{lm}}\left( \text{ϕ}_{\text{ij}} \right)$, which are associated with polar angle $\text{θ}$ and azimuthal angle $\text{ϕ}$ in Eq. (4)*.*

$$\text{q}_{\text{n}\text{l}_{\text{1}}\text{l}_{\text{2}}\text{l}_{\text{3}}}^{\text{i}}\text{=}\sum_{\text{m}_{\text{1}}\text{=}{\text{-}\text{l}}_{\text{1}}}^{\text{l}_{\text{1}}} \sum_{\text{m}_{\text{2}}\text{=}{\text{-}\text{l}}_{\text{2}}}^{\text{l}_{\text{2}}} \sum_{\text{m}_{\text{3}}\text{=}{\text{-}\text{l}}_{\text{3}}}^{\text{l}_{\text{3}}} \left( \begin{matrix} \text{l}_{\text{1}} & \text{l}_{\text{2}} & \text{l}_{\text{3}} \\ \text{m}_{\text{1}} & \text{m}_{\text{2}} & \text{m}_{\text{3}} \end{matrix} \right)\text{×}\text{A}_{\text{n}\text{l}_{\text{1}}\text{m}_{\text{1}}}^{\text{i}}\text{A}_{\text{n}\text{l}_{\text{2}}\text{m}_{\text{2}}}^{\text{i}}\text{A}_{\text{n}\text{l}_{\text{3}}\text{m}_{\text{3}}}^{\text{i}}\text{ }\text{(2)}$$

$$\text{q}_{\text{nl}}^{\text{i}}\text{=}\sum_{\text{m}\text{=-}\text{l}}^{\text{l}} \text{(-1)}^{\text{m}}\text{A}_{\text{nlm}}^{\text{i}}\text{A}_{\text{nl}\text{(-}\text{m}\text{)}}^{\text{i}}\text{ }\text{(3)}$$

Among them:

$$\text{A}_{\text{nlm}}^{\text{i}}\text{=}\sum_{\text{j}\text{≠1}} \text{g}_{\text{n}}\text{(}\text{r}_{\text{ij}}\text{)}\text{Y}_{\text{lm}}\left( \text{θ}_{\text{ij}}\text{,}\text{ϕ}_{\text{ij}} \right)\text{ }\text{(4)}$$

Both radial descriptors and angular descriptors are closely related to the local atomic environment.

## Supplementary Note S6: Initial Training Set Construction and Physics-Guided Active Learning

The entire procedure is designed to systematically achieve comprehensive coverage across the descriptor, configuration, and chemical species spaces, ensuring the accuracy and simulation stability of the final MLP. The initial training set was constructed by combining structural perturbation with a series of AIMD simulations at varying temperatures. Specifically, structural perturbations were performed on equilibrium configurations using the NepTrainKit software^5^, with the force induced by each perturbation constrained to be less than 20 eV/Å to increase configurational diversity within a locally reasonable region of the potential energy surface. Concurrently, AIMD simulations were conducted from 300 K to 900 K with a 200 K increment (2 independent runs per temperature, 10 ps each) to enhance ergodicity and achieve more complete coverage of the high-dimensional descriptor space through elevated-temperature sampling. All AIMD trajectories were monitored in real-time for bond formation/breaking events using the ReaxTools software^6^. For identified key reaction events, dense sampling (at 5 fs intervals) was applied to the trajectory segments immediately before and after the event. These carefully selected configurations were then subjected to DFT single-point calculations to obtain accurate energies, forces, and virials, which were subsequently incorporated into the initial training set.

During the active learning iterations involving MLP-driven molecular dynamics (MLP-MD), a physics-guided correction mechanism was introduced to constrain trajectory exploration and prevent deviations into unphysical regions. The implemented rule is as follows: throughout the MLP-MD simulation, trajectories were output at high frequency and continuously monitored for chemical bond changes using ReaxTools. The emergence of non-physical bonding patterns or precursors to system energy/pressure instability was flagged as an early warning for potential "trajectory collapse." Upon triggering such a warning, the last physically reasonable configuration before the warning point was extracted and used as the initial state to launch a short AIMD simulation. This AIMD segment acts as a "physics corrector," allowing the system to evolve naturally according to the first-principles potential energy surface from that point onward. Key frames along the evolution path from this corrective trajectory, particularly those around transition states crossing energy barriers, were systematically extracted. After DFT calculation, these configurations were added to the training set, thereby using authentic physical reaction pathway information to correct potential biases in the MLP.

The entire workflow forms a closed loop of "sampling–monitoring–correction–training." This process primarily relies on the NepTrainKit tool for initial perturbation, structure screening, and active learning management; utilizes ReaxTools as the core real-time analyzer for chemical environments and reaction events throughout both initial sampling and active learning monitoring phases; and employs custom scripts to automate the iteration by bridging AIMD calculations, configuration extraction, DFT job submission, and data organization. Through the protocols and rules described above, a high-quality training set was constructed, ensuring that the final MLP possesses both broad configurational sampling and high physical fidelity in exploring the potential energy surface for complex interfacial chemical reactions.

## Supplementary Note S7: Training Set Composition

| Compositions | Count | Compositions | Count | Compositions | Count | Compositions | Count |
| --- | --- | --- | --- | --- | --- | --- | --- |
| H144C72O72 | 18 | H40C30O30Na20 | 642 | C336Na33 | 1 | F13Na16 | 11 |
| H120C90O90 | 20 | H44C27O27Na20 | 168 | C288Na32 | 2 | F1Na16 | 2 |
| H210C90O45 | 20 | H54C27O27Na20 | 71 | C144Na6 | 1 | F24Na4P4 | 79 |
| H144C96O72 | 30 | H80C32O16Na20 | 120 | C96Na6 | 2 | F192Na32P32 | 21 |
| H190C76O38 | 25 | H54C36O27Na20 | 36 | C288Na42 | 1 | Na12 | 29 |
| H122C75O75 | 25 | H486C324O243Na180 | 9 | C264Na21 | 2 | Na8 | 50 |
| H190C76O38F12Na2P2 | 16 | H720C288O144Na180 | 3 | C288Na21 | 1 | Na1 | 9 |
| H210C90O45F12Na2P2 | 13 | H630C270O135Na180 | 4 | C192Na9 | 1 | Na2 | 98 |
| H144C96O72F12Na2P2 | 26 | H486C243O243Na180 | 1 | C264Na42 | 2 | Na11 | 7 |
| H210C90O45F24Na4P4 | 16 | H13Na16 | 17 | C240Na15 | 2 | Na4 | 87 |
| H144C96O72F24Na4P4 | 19 | H1Na16 | 11 | C96Na3 | 2 | Na10 | 6 |
| H190C76O38F60Na10P10 | 24 | H8Na16 | 23 | C216Na18 | 2 | Na7 | 7 |
| H122C75O75F60Na10P10 | 29 | C2O6Na4 | 50 | C168Na3 | 1 | Na3 | 6 |
| H210C90O45F60Na10P10 | 16 | C4O12Na8 | 20 | C264Na32 | 1 | Na6 | 59 |
| H144C96O72F60Na10P10 | 20 | C6O18Na6 | 33 | C312Na48 | 1 | Na54 | 4 |
| H190C76O38F120Na20P20 | 18 | C1Na16 | 7 | C360Na33 | 2 | Na9 | 7 |
| H122C75O75F120Na20P20 | 17 | C8Na16 | 33 | C336Na66 | 2 | Na16 | 26 |
| H210C90O45F120Na20P20 | 15 | C13Na16 | 60 | C144Na5 | 1 | Na5 | 5 |
| H144C96O72F120Na20P20 | 18 | C168Na6 | 2 | C360Na66 | 2 | Na35 | 1 |
| H122C75O75F12Na2P2 | 22 | C120Na5 | 1 | C312Na24 | 1 | Na24 | 5 |
| H190C76O38F24Na4P4 | 13 | C120Na6 | 2 | C168Na5 | 1 | Na44 | 2 |
| H122C75O75F24Na4P4 | 26 | C120Na3 | 1 | O13Na16 | 23 | Na14 | 4 |
| H44C27O27F12Na22P2 | 1711 | C216Na12 | 2 | O1Na16 | 8 | Na15 | 2 |
| H70C28O14F12Na22P2 | 265 | C192Na12 | 2 | O8Na16 | 17 | Na53 | 2 |
| H60C24O12F6Na21P1 | 114 | C96Na5 | 1 | O34Na68 | 7 | Na13 | 1 |
| H56C24O12F6Na21P1 | 176 | C144Na3 | 2 | O2Na4 | 15 | Na36 | 2 |
| H32C24O24F12Na22P2 | 7 | C240Na23 | 2 | O3Na6 | 26 | Na16P8 | 19 |
| H42C28O21F12Na22P2 | 78 | C312Na36 | 1 | O6Na12 | 6 | Na16P13 | 16 |
| H288C216O216F108Na198P18 | 6 | C216Na24 | 2 | O6Na6 | 51 | Na16P1 | 7 |
| H378C252O189F108Na198P18 | 10 | C192Na6 | 2 | O6Na36 | 7 | Na6P2 | 17 |
| H504C216O108F54Na189P9 | 2 | C240Na30 | 2 | O6Na3 | 55 | Na8P8 | 10 |
| H540C216O108F54Na189P9 | 3 | C336Na50 | 1 | O4Na8 | 5 | Total | 5035 |
| H70C28O14Na22 | 18 | C360Na50 | 1 | F8Na16 | 15 |  |  |

## Supplementary Note S8: Stability Validation of Large-Scale Simulations

Figure S2. Stability verification of large-scale MLMD simulations (~27,000 atoms).

## Supplementary Note S9: Comparison of Force Prediction Accuracy between ReaxFF and DFT Results.

Figure S3. Parity plots comparing force predictions of ReaxFF and DFT references.

## Supplementary Note S10: DFT- and NEP-calculated Energy Barriers for EC Ring-opening

Figure S4. Comparison of DFT- and NEP-calculated energy barriers for EC ring-opening.

## Supplementary Note S11: Calculation results for the melting point of metallic sodium.

Figure S5. Melting point equilibrium diagram and initial and final structures.

## Supplementary Note S12: Evolution of Reactive Species in Sodium Electrodes with Various Electrolytes

Pure solvent:

Figure S6. Reaction products of pure EC with the sodium metal electrode.

Figure S7. Reaction products of pure PC with the sodium metal electrode.

Figure S8. Reaction products of pure DMC with the sodium metal electrode.

Figure S9. Reaction products of pure DME with the sodium metal electrode.

Figure S10. Reaction products of pure G2 with the sodium metal electrode.

1M NaPF_6_ in solvent:

Figure S11. Reaction products of 1 M NaPF_6_ in EC with the sodium metal electrode.

Figure S12. Reaction products of 1 M NaPF_6_ in PC with the sodium metal electrode.

Figure S13. Reaction products of 1 M NaPF_6_ in DMC with the sodium metal electrode.

Figure S14. Reaction products of 1 M NaPF_6_ in DME with the sodium metal electrode.

Figure S15. Reaction products of 1 M NaPF_6_ in G2 with the sodium metal electrode.

## Supplementary Note S13: Surface Energies of Low-index NaF.

Figure S16. Comparison of surface energies of low-index NaF facets.

## Supplementary Note S14: Thermodynamic Analysis of CO vs CO₂ Formation from EC Decomposition

Figure S17. Gibbs free energy changes (ΔG) for the formation of CO and CO_2_ from EC decomposition at 300 K (DFT-PBE-D3).

## Supplementary Note S15: Comparison of CO and CO_2_ evolution in the EC system during MLMD simulations

Figure S18. Comparison of CO and CO_2_ evolution in the EC system during MLMD simulations at different temperatures.

## Supplementary Note S16: Influence of Concentration on the Composition of Solvated Species

Figure S19. Percentages of various solvation structure types in EC systems with different NaPF_6_ concentrations.

## Supplementary Note S17: Temperature and Concentration Dependence of Electrolyte Transport Properties

Figure S20. (a) Na–O RDFs at different temperatures (260–340 K) for 1 M NaPF_6_ in EC; (b) Corresponding coordination numbers; (c) Na–O RDFs for different concentrations of NaPF_6_ in EC; (d) Corresponding coordination numbers;

As shown in Figure S20a,c, with increasing temperature, the main Na–O radial distribution function (RDF) peak (~2.3 Å) gradually broadens and decreases in height, while the coordination number decreases. This indicates that the binding between Na^+^ and solvent molecules weakens as temperature rises, which typically enhances ion mobility. Consequently, the ionic conductivity exhibits a complex dependence on concentration. At low concentrations, increasing the NaPF_6_ concentration enhances the number of charge carriers, thereby enhancing ionic conductivity. However, at higher concentrations, the significantly strengthened ion-ion interactions (as evidenced by the decrease in the main Na–O peak intensity in Figure S20b,d) conversely reduce ionic conductivity. These trends confirm the transition from SSIP-dominated to AGG-dominated regimes as concentration increases.

## Supplementary Note S18: Role of Aggregates (AGGs) in Non-Uniform SEI Growth

Figure S21. Reaction structural evolution in the large-scale Na-EC system at 350 K.

## Supplementary Note S19: Well-Tempered Metadynamics Simulations

**Theoretical Foundation of Well-Tempered Metadynamics**

In a typical metadynamics simulation, an external history-dependent bias potential is gradually introduced in the space defined by selected collective variables (CVs) at specific time intervals. This history-dependent potential, generally expressed as a sum of Gaussian functions, can be formulated as follows^7,8^:

$$V\left( \vec{s},t \right)=\sum_{k\tau<t} W\left( k\tau\right)\exp\left( -\sum_{i=1}^{d} \frac{\left[ s_{i}-s_{i}\left( q\left( k\tau\right) \right) \right]^{2}}{2\sigma_{i}^{2}} \right)$$

where $\tau, W\left( k\tau\right)$, and $\sigma_{i}$ denote the Gaussian deposition stride, the height of the Gaussian function, and the width along the CVs, respectively. By introducing the metadynamics bias potential, this approach ensures that the system is driven away from local minima, enabling it to explore the entire phase space and reconstruct the free energy surface.

Metadynamics techniques have been successfully employed to elucidate atomic-scale interactions at interfaces, including solid/liquid, liquid/liquid, and liquid/vacuum interfaces. These interfaces play crucial roles in governing molecular reactions across diverse fields such as catalysis, energy storage, and environmental science^7,9,10^.

**Metadynamic simulation details**

The sodium storage process simulations were performed using the GPUMD package integrated with the PLUMED plugin to construct the free energy landscape during deposition. The following sections describe the overall workflow of the deposition cycle, the setup of metadynamics parameters, and the method for free energy calculation.

A cyclic deposition strategy was adopted, where each deposition cycle consisted of three stages: structural initialization, molecular dynamics relaxation, and metadynamics sampling. The initial model comprised an electrode interface and an electrolyte environment. At the beginning of each cycle, a single sodium ion (in the form of a Na^+^-solvation cluster) was introduced into the surface region of the system. The ion position was adjusted to maintain a suitable distance from the surface, avoiding initial overlap. Subsequently, pre-equilibrium was carried out in the canonical ensemble (NVT) at 350 K.

In the metadynamics simulations, the axial distance of the sodium ion along the surface normal direction was chosen as the collective variable (CV). A reference point was fixed approximately 2 Å above the carbon layer of the electrode surface. The CV was defined as the projected distance in the z-direction between the sodium ion and this reference point. To restrict lateral diffusion of the ion, a soft wall potential was applied to confine its horizontal displacement, ensuring that sampling occurred predominantly along the direction perpendicular to the interface. Furthermore, lower and upper boundaries were imposed in the CV space to confine the sampling window near the interface, thereby improving sampling efficiency.

The Well-Tempered Metadynamics (WT-MetaD) method was employed to apply the bias potential. Gaussian hills with heights of 0.01 eV (initial phase) and 0.0001 eV (equilibrium phase), a width of 0.20 Å, and a deposition stride of 100 steps were used. The bias factor was set to 80, and the simulation temperature was maintained at 300 K. This approach adaptively fills the free energy basins, facilitating ion translocation over energy barriers and enabling efficient phase space sampling. To improve convergence, the simulation was divided into two stages: an initial medium bias strength for rapid exploration of the barrier region, followed by a reduced bias magnitude for refined sampling. The simulations employed a neuroevolution potential (NEP) to describe interatomic interactions, with a time step of 0.5 fs. Each metadynamics segment lasted approximately 100 ps, and a total of 100 deposition cycles were performed. After each cycle, the final configuration was retained as the initial structure for the subsequent cycle, enabling the simulation of continuous sodium ion deposition. The evolution of collective variables and the convergence of the bias potential were analyzed using the COLVAR and HILLS files generated by PLUMED. Finally, the free energy surface was reconstructed using reweighting techniques to evaluate the thermodynamic stability of sodium ions at various interfacial positions.

## Supplementary Note S20: Structural Evolution of the Substrate during Sodium Storage

Figure S22. Structural evolution of the Na_2_CO_3_-EC system during charging

Figure S23. Structural evolution of the NaF-DME system during charging.

## Supplementary Note S21: Evolution of Electrolyte Consumption during the Charging Process.

Figure S24. Evolution of electrolyte consumption during the charging process.

## Supplementary Note S22: Effect of Pore Size and Spatial Confinement on Sodium Storage

Figure S25. Charge states of sodium storage in carbon nanotubes with different diameters.

In addition to the interfacial chemistry, the physical storage environment within the carbon anode significantly influences the sodium storage mechanism. To investigate the effect of spatial confinement, we modeled sodium storage in carbon-based anodes with varying pore sizes (Figure S1).

The storage morphology of sodium is found to be strongly dependent on the spatial confinement effects of the host material. For confined spaces (e.g., narrow pores), the electrons of the sodium atoms in the layer directly contacting the carbon wall tend to be localized, forming ionic bonds with carbon. This results in a distinct ionic character for the stored sodium (Figure S1a). However, when the storage space allows for multi-layer stacking of sodium atoms (e.g., with a pore size of 11.75 Å as shown in Figure S1b), the inner sodium atoms that are not in direct contact with the carbon substrate display characteristics of quasi-metallic sodium. These atoms retain their metallic nature and exhibit charge properties closer to bulk sodium.

This result indicates a size-dependent transition in the sodium storage mechanism: as the pore size increases, the dominant mechanism shifts from interface-adsorption-dominated ionic storage to bulk-filling-dominated quasi-metallic storage. These findings highlight that optimizing the pore structure of carbon anodes is crucial for maximizing the capacity of metallic sodium deposition versus surface adsorption.

## Supplementary Note S23: X-ray Photoelectron Spectroscopy (XPS) Measurements.

X-ray photoelectron spectroscopy (XPS) measurements were performed using a Thermo Scientific K-Alpha X-ray photoelectron spectrometer. The sample was an electrode prepared by reacting a sodium metal-loaded copper sheet with electrolyte after one day of static storage, aiming to analyze the chemical composition and element valence states of the solid electrolyte interphase (SEI) on its surface. The sample was cut and prepared in an inert atmosphere glovebox to prevent surface oxidation or contamination from external factors. During testing, a monochromatic Al Kα X-ray source (hv = 1486.6 eV) was employed with a power of approximately 150 W and a spot size of about 400 μm. The binding energy was calibrated using the C 1s peak (284.8 eV). The measurements included full-spectrum scanning (0–1350 eV) and high-resolution scans of core-level orbitals such as O 1s, Na 1s, P 2p, C 1s, and F 1s. The obtained data were preliminarily processed using Thermo Avantage software, including background subtraction, peak fitting, and semi-quantitative analysis.

## Supplementary Note S24: Gas Chromatography (GC) Measurements.

The qualitative and quantitative analysis of the reaction-generated gases was conducted using a custom online gas chromatography (GC) system. All preparation and assembly were performed in an argon-filled glovebox (H_2_O and O_2_ < 0.1 ppm). The sodium metal pieces were sealed in a dry 10 mL glass vial, which was then connected to a flow system outside the glovebox. The system, maintained under a positive pressure of high-purity argon (>99.999%), comprised a T-union with one port for continuous argon supply, one sealed with a rubber septum for injection, and the outlet connected to the GC (HF-901, Shandong Huifen Instrument) injection port. Before reaction, the entire flow path was purged with argon (50 mL min^–1^) for 5 min. Then, 5.0 mL of electrolyte (1 M NaPF_6_ in EC or DME) was injected through the septum to contact the sodium metal. The resulting gases were carried by the argon stream directly into the GC for analysis.

## Supplementary Note S25: First-Principles Evidence for the Self-Passivating Character of the NaF Layer

To directly substantiate the self-passivating, self-limiting role of the NaF layer discussed in the main text (Fig. 4), first-principles calculations were performed to quantify the Na^+^ migration barrier and the Na vacancy formation energy in crystalline NaF, from which the intrinsic Na^+^ conductivity is derived. The calculations were carried out using density functional theory as implemented in VASP, adopting the same calculation setup as in Supplementary Note S3.

Na^+^ migration barrier (E_m_). The migration barrier for a Na^+^ hopping to its nearest-neighbor Na vacancy was computed using the climbing-image nudged elastic band (CI-NEB) method in a 3×3×3 NaF supercell, with size convergence verified. The calculated migration barrier is E_m_ = 0.74 eV (Figure S26).

Na vacancy formation energy (E_f_). The Na vacancy formation energy was evaluated under the Na-rich limit, taking the Na metal electrode as the chemical-potential reservoir. The formation energy is as high as E_f_ = 6.01 eV, indicating that Na^+^ is extremely stable in the ionic NaF lattice and that vacancy formation is highly unfavorable.

Intrinsic vacancy concentration and ionic conductivity. Combining the two quantities, the overall activation energy for Na^+^ transport is E_a_ = E_f_ + E_m_ = 6.76 eV. At 300 K, this corresponds to an intrinsic Na vacancy concentration on the order of ~10^−101^ and an intrinsic Na^+^ conductivity of only ~10^−110^ S cm^−1^ (Figure S27). These results directly demonstrate that Na^+^ can hardly traverse the NaF layer, thereby blocking sustained contact between Na^+^ and the electrolyte and physically confirming the self-passivating, self-limiting character of the NaF layer against subsequent solvent decomposition.

Figure S26. CI-NEB migration barrier of Na^+^ in NaF (E_m_ = 0.74 eV).

Figure S27. Comparison of the Na vacancy formation energy (E_f_), migration barrier (E_m_), and overall activation energy (E_a_ = 6.76 eV), together with the intrinsic Na^+^ conductivity of NaF as a function of temperature.

## Supplementary Note S26: XPS Characterization of the SEI after Charging

To partially bridge the gap between the static SEI characterizations (Fig. 5) and the dynamic SEI formation revealed by our simulations, we additionally performed XPS characterization of the SEI formed after a simple charging step (deposition of a defined amount of metallic Na), using the same XPS setup as described in Supplementary Note S23. The resulting C1s and O1s spectra for both the Na-EC and Na-DME systems are shown in Figure S28.

Figure S28. XPS characterization of the SEI formed after a charging step. (a) C1s in Na-EC system; (b) O1s in Na-EC system; (c) C1s in Na-DME system; (d) O1s in Na-DME system.

## Supplementary Note S27: Desolvation Barrier and Charge-Distribution Analyses of Na Deposition

To rationalize the distinct Na deposition behaviors at the NaF- and Na_2_CO_3_-dominated SEI interfaces (Fig. 7), we performed two additional analyses.

Na^+^ desolvation barrier. The Na^+^ desolvation barriers of the Na–DME and Na–EC groups were evaluated by DFT calculations as the energy difference between the solvated Na^+^ complex and the desolvated state, using the same VASP setup as in Supplementary Note S3. The Na–DME group exhibits a much lower desolvation barrier (+0.447 eV) than the Na–EC group (+2.110 eV) (Figure S30), providing a thermodynamic basis for the distinct initial Na^+^ deposition at the two SEI interfaces.

Evolution of the Na ionic charge distribution. The evolution of the Na ionic charge-density distribution over time (Figure S29) confirms the solid-solution-then-deposition mechanism in the NaF system. Owing to the more ionic character of sodium in NaF, the matrix exhibits a higher charge loss in the early stage and incorporates a small amount of Na^+^ to compensate the charge; because of the low solid-solubility of NaF, the matrix quickly reaches saturation, after which subsequently arriving Na^+^ deposits directly in the metallic state.

Figure S29. Evolution of the Na ionic charge-density distribution over time, illustrating the “solid-solution-then-deposition” mechanism in the NaF system.

Figure S30. Computed Na^+^ desolvation barriers for the Na–DME and Na–EC groups (+0.447 eV vs. +2.110 eV).

**Figure S31.** Elemental-distribution maps (F, H, P, O, C, Na) of the SEI in the Na–EC system, showing random/mixed growth.

**Figure S32.** Quantitative reaction analysis of the large system (Na-DME), illustrating the complexity of the reactions.

**Figure S33.** Quantitative reaction analysis of the large system (Na-EC).

## REFERENCES

(1)	Fan, Z.; Wang, Y.; Ying, P.; Song, K.; Wang, J.; Wang, Y.; Zeng, Z.; Xu, K.; Lindgren, E.; Rahm, J. M.; Gabourie, A. J.; Liu, J.; Dong, H.; Wu, J.; Chen, Y.; Zhong, Z.; Sun, J.; Erhart, P.; Su, Y.; Ala-Nissila, T. GPUMD: A Package for Constructing Accurate Machine-Learned Potentials and Performing Highly Efficient Atomistic Simulations. *J. Chem. Phys.* **2022**, *157* (11), 114801. https://doi.org/10.1063/5.0106617.

(2)	Xu, K.; Bu, H.; Pan, S.; Lindgren, E.; Wu, Y.; Wang, Y.; Liu, J.; Song, K.; Xu, B.; Li, Y.; Hainer, T.; Svensson, L.; Wiktor, J.; Zhao, R.; Huang, H.; Qian, C.; Zhang, S.; Zeng, Z.; Zhang, B.; Tang, B.; Xiao, Y.; Yan, Z.; Shi, J.; Liang, Z.; Wang, J.; Liang, T.; Cao, S.; Wang, Y.; Ying, P.; Xu, N.; Chen, C.; Zhang, Y.; Chen, Z.; Wu, X.; Jiang, W.; Berger, E.; Li, Y.; Chen, S.; Gabourie, A. J.; Dong, H.; Xiong, S.; Wei, N.; Chen, Y.; Xu, J.; Ding, F.; Sun, Z.; Ala‐Nissila, T.; Harju, A.; Zheng, J.; Guan, P.; Erhart, P.; Sun, J.; Ouyang, W.; Su, Y.; Fan, Z. GPUMD 4.0: A High‐performance Molecular Dynamics Package for Versatile Materials Simulations with Machine‐learned Potentials. *Mater. Genome Eng. Adv.* **2025**, e70028. https://doi.org/10.1002/mgea.70028.

(3)	Song, Z.; Han, J.; Henkelman, G.; Li, L. Charge-Optimized Electrostatic Interaction Atom-Centered Neural Network Algorithm. *J. Chem. Theory Comput.* **2024**, *20* (5), 2088–2097. https://doi.org/10.1021/acs.jctc.3c01254.

(4)	Schaul, T.; Glasmachers, T.; Schmidhuber, J. High Dimensions and Heavy Tails for Natural Evolution Strategies. In *Proceedings of the 13th Annual Conference on Genetic and Evolutionary Computation*; GECCO ’11; Association for Computing Machinery: New York, NY, USA, 2011; pp 845–852. https://doi.org/10.1145/2001576.2001692.

(5)	Chen, C.; Li, Y.; Zhao, R.; Liu, Z.; Fan, Z.; Tang, G.; Wang, Z. NepTrain and NepTrainKit: Automated Active Learning and Visualization Toolkit for Neuroevolution Potentials. *Comput. Phys. Commun.* **2025**, *317*, 109859. https://doi.org/10.1016/j.cpc.2025.109859.

(6)	Chen, H. ReaxTools: A High Performance ReaxFF/AIMD/MLP-MD Post-Process Code (Computer Software), 2025. https://github.com/tgraphite/reax_tools (accessed 2025-05-01).

(7)	Ming, X.; Si, W.; Yu, Q.; Sun, Z.; Qiu, G.; Cao, M.; Li, Y.; Li, Z. Molecular Insight into the Initial Hydration of Tricalcium Aluminate. *Nat. Commun.* **2024**, *15* (1), 2929. https://doi.org/10.1038/s41467-024-47164-0.

(8)	Réocreux, R.; Girel, É.; Clabaut, P.; Tuel, A.; Besson, M.; Chaumonnot, A.; Cabiac, A.; Sautet, P.; Michel, C. Reactivity of Shape-Controlled Crystals and Metadynamics Simulations Locate the Weak Spots of Alumina in Water. *Nat. Commun.* **2019**, *10* (1), 3139. https://doi.org/10.1038/s41467-019-10981-9.

(9)	Zhang, Y.; Huang, H.; Tian, J.; Li, C.; Jiang, Y.; Fan, Z.; Pan, L. Modelling Electrified Microporous Carbon/Electrolyte Electrochemical Interface and Unraveling Charge Storage Mechanism by Machine Learning Accelerated Molecular Dynamics. *Energy Storage Mater.* **2023**, *63*, 103069. https://doi.org/10.1016/j.ensm.2023.103069.

(10)	Li, Y.; Pan, H.; Li, Z. Unravelling the Dissolution Dynamics of Silicate Minerals by Deep Learning Molecular Dynamics Simulation: A Case of Dicalcium Silicate. *Cem. Concr. Res.* **2023**, *165*, 107092. https://doi.org/10.1016/j.cemconres.2023.107092.
